# Effect of Two Models of Intrauterine Growth Restriction on Alveolarization in Rat Lungs: Morphometric and Gene Expression Analysis

**DOI:** 10.1371/journal.pone.0078326

**Published:** 2013-11-21

**Authors:** Elodie Zana-Taieb, Laura Butruille, Marie-Laure Franco-Montoya, Emmanuel Lopez, Flore Vernier, Isabelle Grandvuillemin, Danièle Evain-Brion, Philippe Deruelle, Olivier Baud, Christophe Delacourt, Pierre-Henri Jarreau

**Affiliations:** 1 Institut National de la Santé Et de la Recherche Médicale (INSERM) U767, Paris, France; 2 PremUp, Paris, France; 3 Service de Médecine et Réanimation néonatales de Port-Royal, Groupe hospitalier Cochin, Broca, Hôtel-Dieu, Assistance Publique – Hôpitaux de Paris, Paris, France; 4 Unité environnement périnatal et croissance, EA4489, Faculté de Médecine, Pôle recherche, IFR 114,Université Lille Nord de France, Lille, France; 5 Institut National de la Santé Et de la Recherche Médicale (INSERM) U955 IMRB Equipe 04, Créteil, France; 6 Institut National de la Santé Et de la Recherche Médicale (INSERM) UMR 1076, Faculté de Pharmacie, Université de la Méditerranée. Marseille, France; 7 Institut National de la Santé Et de la Recherche Médicale (INSERM) U676, Paris, France; 8 Service de Pneumologie Pédiatrique, Hôpital Necker-Enfants Malades, Assistance Publique – Hôpitaux de Paris, Paris, France; 9 Université Paris Descartes, Paris, France; 10 Service de Réanimation et Pédiatrie néonatales, Hôpital Robert Debré, Assistance Publique – Hôpitaux de Paris, Paris, France; 11 Université Paris Diderot, Paris, France; University of North Carolina School of Medicine, United States of America

## Abstract

Intrauterine growth restriction (IUGR) in preterm infants increases the risk of bronchopulmonary dysplasia, characterized by arrested alveolarization. We evaluated the impact of two different rat models (nitric oxide synthase inhibition or protein deprivation) of IUGR on alveolarization, before, during, and at the end of this postnatal process. We studied IUGR rat pups of dams fed either a low protein (LPD) or a normal diet throughout gestation and pups of dams treated by continuous infusion of Nω-nitro-L-arginine methyl ester (L-NAME) or its diluent on the last four days of gestation. Morphometric parameters, alveolar surface (Svap), mean linear intercept (MLI) and radial alveolar count (RAC) and transcriptomic analysis were determined with special focus on genes involved in alveolarization. IUGR pups regained normal weight at day 21 in the two treated groups. In the LPD group, Svap, MLI and RAC were not different from those of controls at day 4, but were significantly decreased at day 21, indicating alveolarization arrest. In the L-NAME group, Svap and RAC were significantly decreased and MLI was increased at day 4 with complete correction at day 21. In the L-NAME model, several factors involved in alveolarization, VEGF, VEGF-R1 and –R2, MMP14, MMP16, FGFR3 and 4, FGF18 and 7, were significantly decreased at day 4 and/or day 10, while the various factors studied were not modified in the LPD group. These results demonstrate that only maternal protein deprivation leads to sustained impairment of alveolarization in rat pups, whereas L-NAME impairs lung development before alveolarization. Known growth factors involved in lung development do not seem to be involved in LPD-induced alveolarization disorders, raising the question of a possible programming of altered alveolarization.

## Introduction

Bronchopulmonary dysplasia (BPD) is a chronic lung disease affecting preterm infants and characterized by arrested alveolarization in the developmental program. Lungs affected by BPD exhibit fewer and larger alveoli and decreased pulmonary microvascular development [1]. Several recent reports indicate that intrauterine growth restriction (IUGR), defined as failure of the fetus to achieve the expected weight for a given gestational age [2], could contribute to the development of BPD [3]. Most cases of IUGR are due to placental insufficiency, which decreases placental and umbilical blood flow and therefore placental transfer of glucose, essential amino acids and oxygen to the fetus [4]. IUGR is an important health problem, responsible for an increased risk of morbidity and perinatal mortality [4] and has long-term consequences, such as metabolic syndrome, usually attributed to “fetal programming”. Some studies have also suggested that compromised fetal growth can lead to lung dysfunction during infancy [3], [5], childhood [5], [6], and adulthood [7], [8]. However, the mechanistic basis for a relationship between IUGR and BPD and the possibility of fetal programming of lung development have not been elucidated, as the effects of experimental models of IUGR on lung development depend on the model studied, especially timing, nature of the insult and species [9].

Various models affecting the quality of intrauterine environment and leading to IUGR have been developed, such as low-protein diet (LPD), uterine artery ligation, administration of N-omega-nitro-L-arginine methyl ester (L-NAME, a non-specific NO synthase inhibitor), and gestational hyperoxia or hypoxia [10]. The effects of these models on lung development have not been previously reported. Diaz *et al.* in a model of IUGR induced by L-NAME, suggested that postnatal catch-up growth may completely correct lung development disorders present at birth in IUGR rat pups, [11], whereas Maritz *et al.*, inducing IUGR by umbilico-placental embolization in sheep, showed that structural alterations in the lung induced by IUGR were apparent at 8 weeks and still present 2 years after birth, indicating that IUGR may result in permanent changes [12], [13].

To investigate the relationship between IUGR and alveolarization, a key feature of BPD, we studied two models of IUGR in rodents: one induced by maternal protein restriction throughout gestation [10], [14] and the other induced by L-NAME from day 17 until the end of gestation [15]. Morphometric analysis was performed on lung tissue from rat pups at three key time-points of alveolar development in the rat, from postnatal day 4 (P4) to P21, a period characterized by a sharp rise in gas exchange surface area [16]. Alveologenesis is a highly integrated process that implies cooperative interactions between interstitial, epithelial, and vascular compartments of the lung involving several key control-molecules as various transcription factors, growth factors and matrix-remodeling enzymes [16]. To study the molecular basis of IUGR-induced altered alveolarization in these two models, gene expression analysis for 13 key genes involved in alveolarization and angiogenesis was therefore performed on P10.

Our results demonstrate that only maternal protein deprivation leads to sustained impairment of alveolarization in rat pups, whereas L-NAME impairs lung development only before alveolarization, with full recovery thereafter at P21. Known factors of lung development do not seem to be involved in IUGR-induced alveolarization disorders in either of the two models.

## Materials and Methods

### Animals and diets

All experiments were carried out in compliance with INSERM ethical rules and the recommendations of the National Research Council's Guide for the Care and Use of Laboratory Animals. Experiments were designed to minimize the number of animals needed and the discomfort during experimental procedures. On days 4, 10 and 21, rat pups were killed by an intraperitoneal overdose of pentobarbital sodium (70 µg/g body weight) and then exsanguinated by aortic transection. The “Charles River Laboratories Ethics Committee” (10/17/11 and 07/28/12) approved the low-protein diet protocol. French Ministry of Agriculture animal use accreditation (no. 04860) has been granted to the DHURE laboratory in Lille for experimentation with rats.

Female Sprague Dawley rats from Charles River (l'Abresle, France) for the LPD model and Janvier (LeGenest St Isle, France) for the L-NAME model were mated with a male. Day 1 of pregnancy (E1) was determined by the presence of spermatozoa in vaginal smears. Pregnant rats were housed individually with free access to food and water under cyclic controlled light. All dams littered spontaneously. Day of birth was defined as P0. At P4, P10 and P21, pups were weighed before being killed and their lungs were harvested for determination of morphometric parameters or were frozen.

#### LPD-induced IUGR

Dams were randomly divided into two groups and fed with different diets from the day of conception until P2. A 22% protein diet was used for the Control group, and an isocaloric 9% protein diet was used for the LPD group [14]. Caloric deficiency in the LPD group was compensated by carbohydrates. After birth, litters were equalized to 10 pups.

#### L-NAME-induced IUGR

Dams were fed with normal diet and, on day 17 of gestation, randomly assigned to receive L-NAME or saline until the end of gestation. An Alzet osmotic pump (Direct Corp., Palo Alto, CA) was placed subcutaneously on the rat's back after general anesthesia with Isoflurane® and was used to infuse either L-NAME (SIGMA ALDRICH N5751, 98% powder diluted to a final dilution of 50 mg/dL) or saline vehicle. The infusion continued until the end of gestation. To avoid a potential effect of the product delivered via breast milk, equalized litters of 10 pups were adopted on P2 by dams free of any treatment or pump.

### Lung analysis

#### Morphometry

A cannula adapted to the size of the trachea was selected. The cannula, filled with 4% paraformaldehyde (PFA), was inserted into the trachea and used to inflate the lungs with PFA at a pressure of 20 cmH_2_O. Lungs were then kept inflated by tracheal occlusion and lung volume was evaluated using the fluid displacement method [17]. Lung volume was calculated on the basis of the measured weight increase and the specific gravity of PFA (∼1 g/cm3). Five animals selected at random from each group were studied at each time-point.

Alveolar surface density (Sv(a,p)) was determined using point counting and mean linear intercept (MLI) methods described by Weibel [18]. Absolute surface area (Sa) per lung was calculated by multiplying surface density by lung volume. Radial alveolar count (RAC) was performed according to Emery's method [19].

#### Quantitative real-time PCR

mRNA expression of genes closely associated with alveolarization i.e. PDGF-A, MMP14 and 16, FGF7 and 18 and their receptors FGFR3 and 4 and angiogenesis i.e. Tie 1 and 2, VEGF and its receptors, VEGFR1 and 2, and adrenomedullin (ADM) were quantified at P4, P10 and P21. Total RNA was extracted from the entire lung tissue using RNeasy Mini RNA extraction kit (Qiagen, Chatsworth, CA). Purified RNA was quantified using NanoDrop™ Spectrophotometer technology (Thermo Fischer Scientific, Wilmington, Delaware). Total RNA was converted into cDNA using 500 ng of total RNA, Superscript III reverse transcriptase, and random hexamer primers (Invitrogen). Real-time PCR was performed on an ABI Prism 7000 (Applied Biosystems, Courtaboeuf, France) using the following protocol: initial denaturation (10 min at 95°C), then a two-step amplification program (15 s at 95°C followed by 1 min at 60°C) repeated 40 times, and finally a dissociation process. Each reaction consisted of a cDNA equivalent of 0.5 µg of total RNA, 12.5 µl SYBR Green PCR Master Mix and forward and 0.9 or 0.3 mM of reverse primers in a 25 µl reaction volume. Levels of mRNA expression were normalized to a housekeeping gene (18 s mRNA) and expressed as relative value using the ΔΔCt method relative to a calibrator sample.

Primers were designed using Primer Express 3.0 (Applied Biosystems, Foster city, CA). Primer pairs are listed in Table S1.

### Statistical analysis

Multiple group comparisons were performed by Kruskal-Wallis analysis, and two-group comparisons were performed by Mann-Whitney U test using GraphPad Prism software (GraphPad software INC, San Diego, CA). A p value<0.05 was considered significant.

## Results

### Weight and growth

Body weight was significantly lower in the LPD group compared to the control group on P4 and P10, but not on P21 with total catch-up of growth (Table 1). Body weight was also significantly lower in the L-NAME group (Table 2) on P4 and P10 and was slightly higher than that of the control group on P21.

**Table 1:** Weight gain: at P4, P10 and P21 of rat pups in the low protein diet-induced intrauterine growth restriction group and control.

	P4		P10		P21	
	Control	LPD	Control	LPD	Control	LPD
Weight (g)	10.6±1.17	7.6±0.74*	22.3±1.01	17.9±1.64*	47.2±2.15	48.4±2.56

Values are expressed as mean ± SEM.

* p<0.05 between control and LPD group; two-tailed Mann-Whitney test. n = 16–20 rat pups per group.

**Table 2:** Weight gain: at P4, P10 and P21 of rat pups in L-NAME-induced intrauterine growth restriction group and control.

	P4		P10		P21	
	Control	L-NAME	Control	L-NAME	Control	L-NAME
Weight (g)	10.8±0.47	8.6±0.87*	22.1±1.05	19.6±1.41*	40.9±4.44	45.8±3.9*

The significance for each bar is indicated by *p<0.05, control vs. L-NAME; two-tailed Mann-Whitney test. Values are expressed as mean ± SEM. n = 5–10 rat pups per group.

### Morphometric analysis

**Figure 1:**
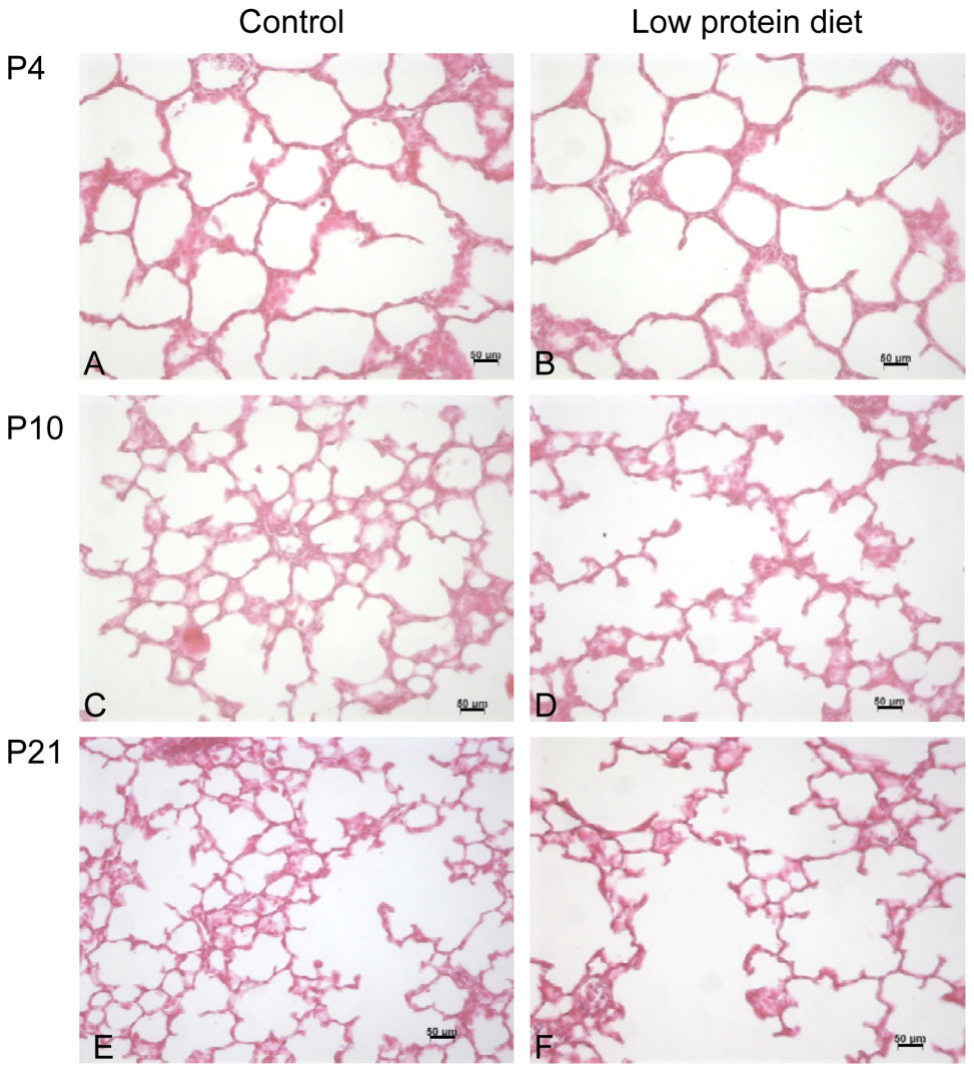
Light microscopic appearance of the lung in control and low protein diet rat pups at P4 (A and B), P10 (C and D), and P21 (E and F) of postnatal life. Photographs of the alveolar region, taken at the same magnification, are presented for each group.

**Figure 2:**
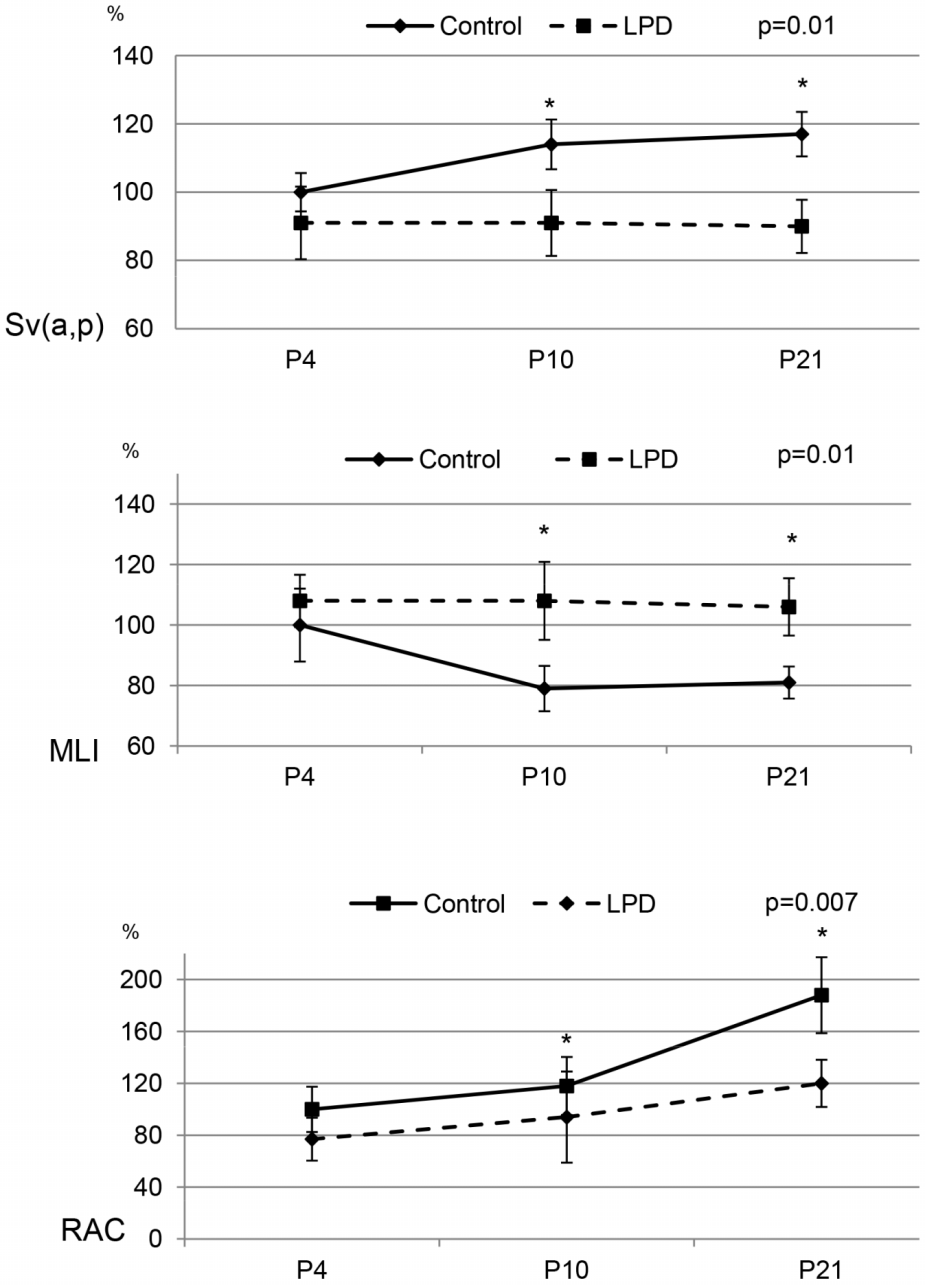
Morphometric analysis in control and low protein diet rat pups. Sv(a,p), Mean Linear Intercept (MLI) and Radial alveolar count (RAC) at P4, P10, and P21 were expressed as percentage of mean control value. Significance for each bar is indicated by p values, Control vs. LPD; two-tailed Mann-Whitney test (p<0.05). N = 5 animals per group.

Maternal LPD resulted in significantly decreased alveolarization on P21 (Fig. 1), at the end of the process, resulting in simplified lung structure with enlarged airspaces and fewer secondary septa, whereas no difference was observed on P4 (Fig. 1 and Table S2). Morphometric analysis showed a significant 30% increase of MLI on P10 and P21 and a significant decrease of Sv(a,p) in the same proportions, while no significant difference was observed on P4 in the LPD group compared to controls. On P21, RAC was decreased by 35% in the LPD group (Fig. 2).

**Figure 3:**
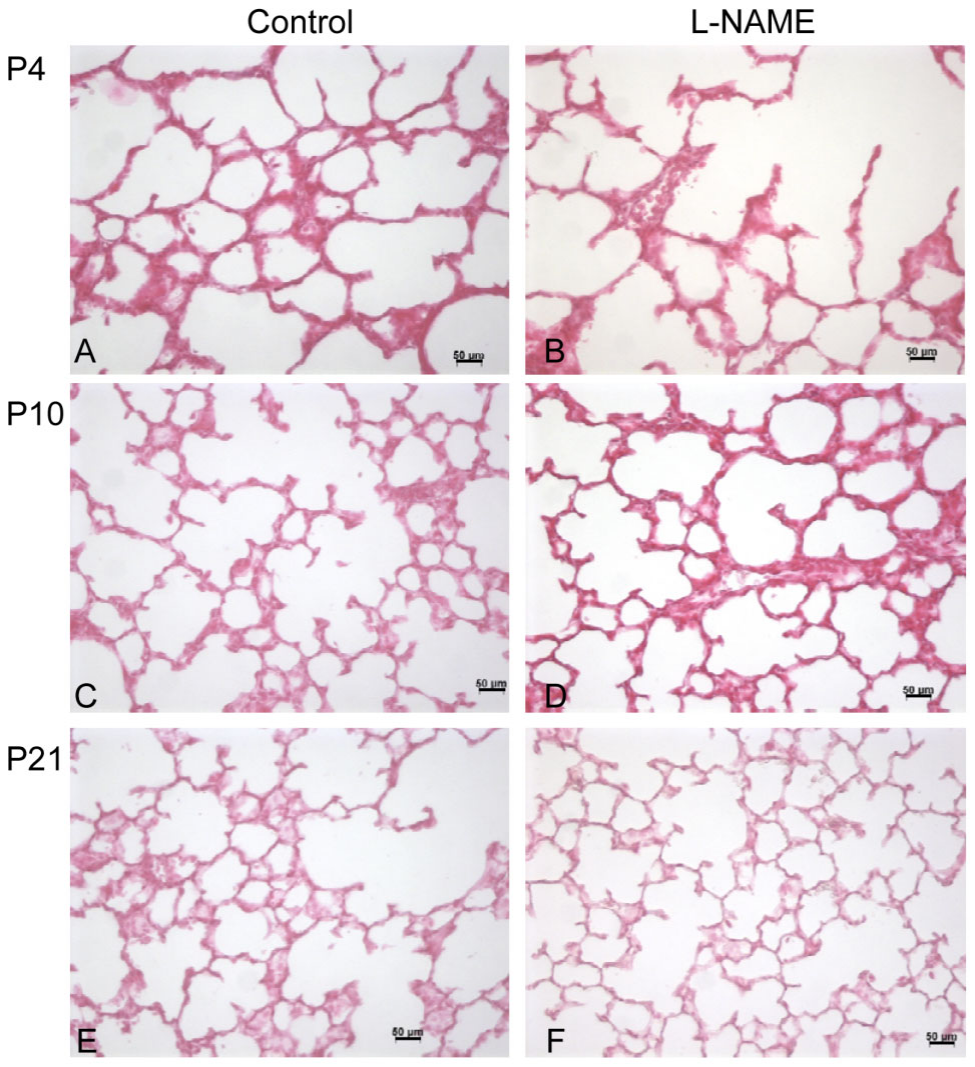
Light microscopic appearance of the lung in control and L-NAME rat pups at P4 (A and B), P10 (C and D), and P21 (E and F) of postnatal life. Photographs of the alveolar region, taken at the same magnification, are presented for each group.

**Figure 4:**
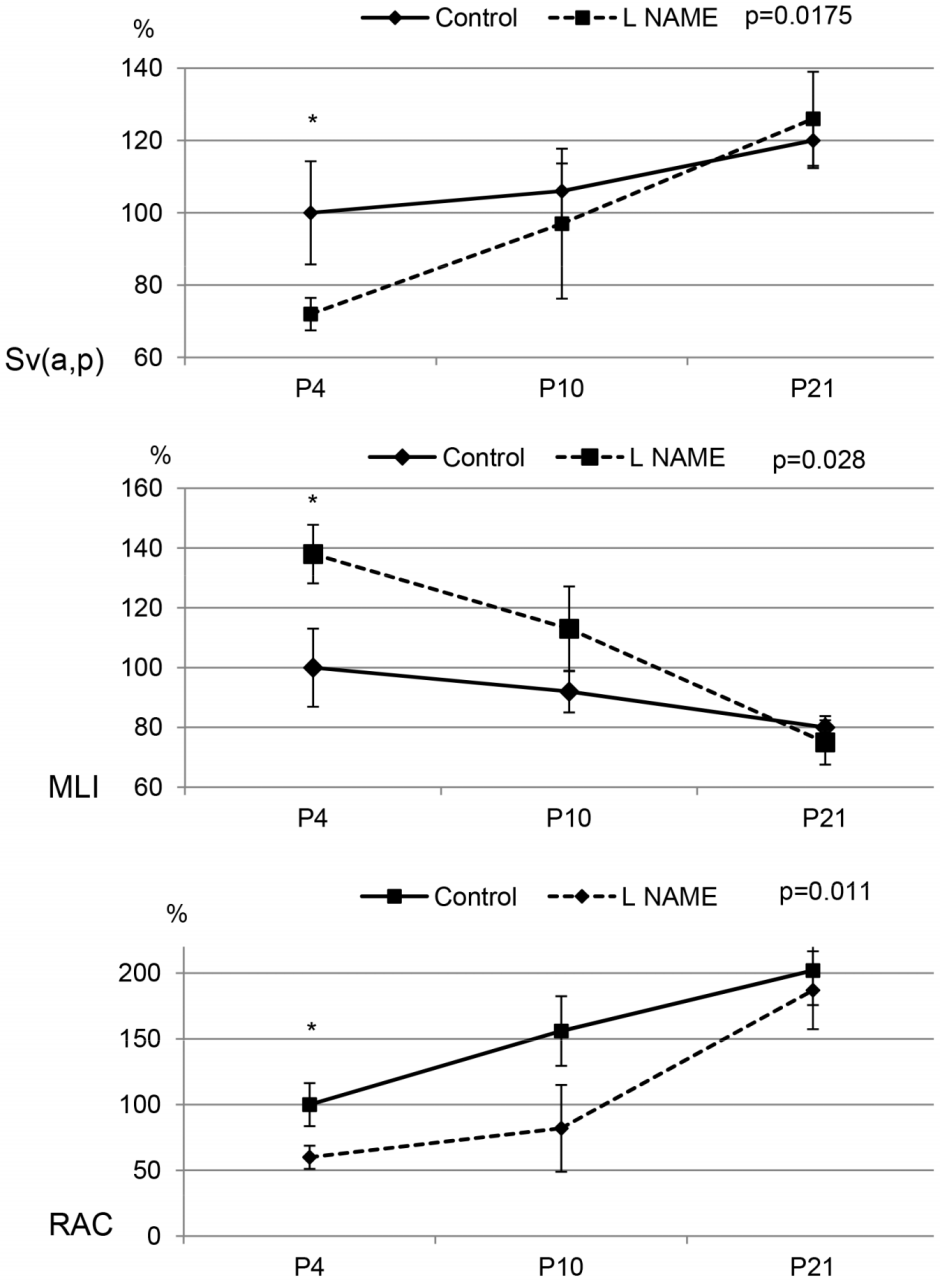
Morphometric analysis in control and L-NAME rat pups. Sv(a,p), Mean Linear Intercept (MLI) and Radial alveolar count (RAC) in control and L-NAME rat pups at P4, P10, and P21 were expressed as percentage of mean control value.. The significance for each time-point is indicated by p values, control vs. L-NAME; two-tailed Mann-Whitney test (p<0.05). n = 5 animals per group.

Maternal injection of L-NAME resulted in significantly decreased alveolarization on P4, at the beginning of the alveolarization process, but not at P21. Lungs from animals exposed to L-NAME exhibited enlarged alveoli on P4, but not on P10 and P21 (Fig. 3 and Table S3). Mean linear intercept was increased by 60% and Sv(a,p) and RAC was decreased in the same proportion on P4, while no between-group differences were observed thereafter (Fig. 4).

### PCR results

#### Gene expression in LPD-induced IUGR

**Figure 5:**
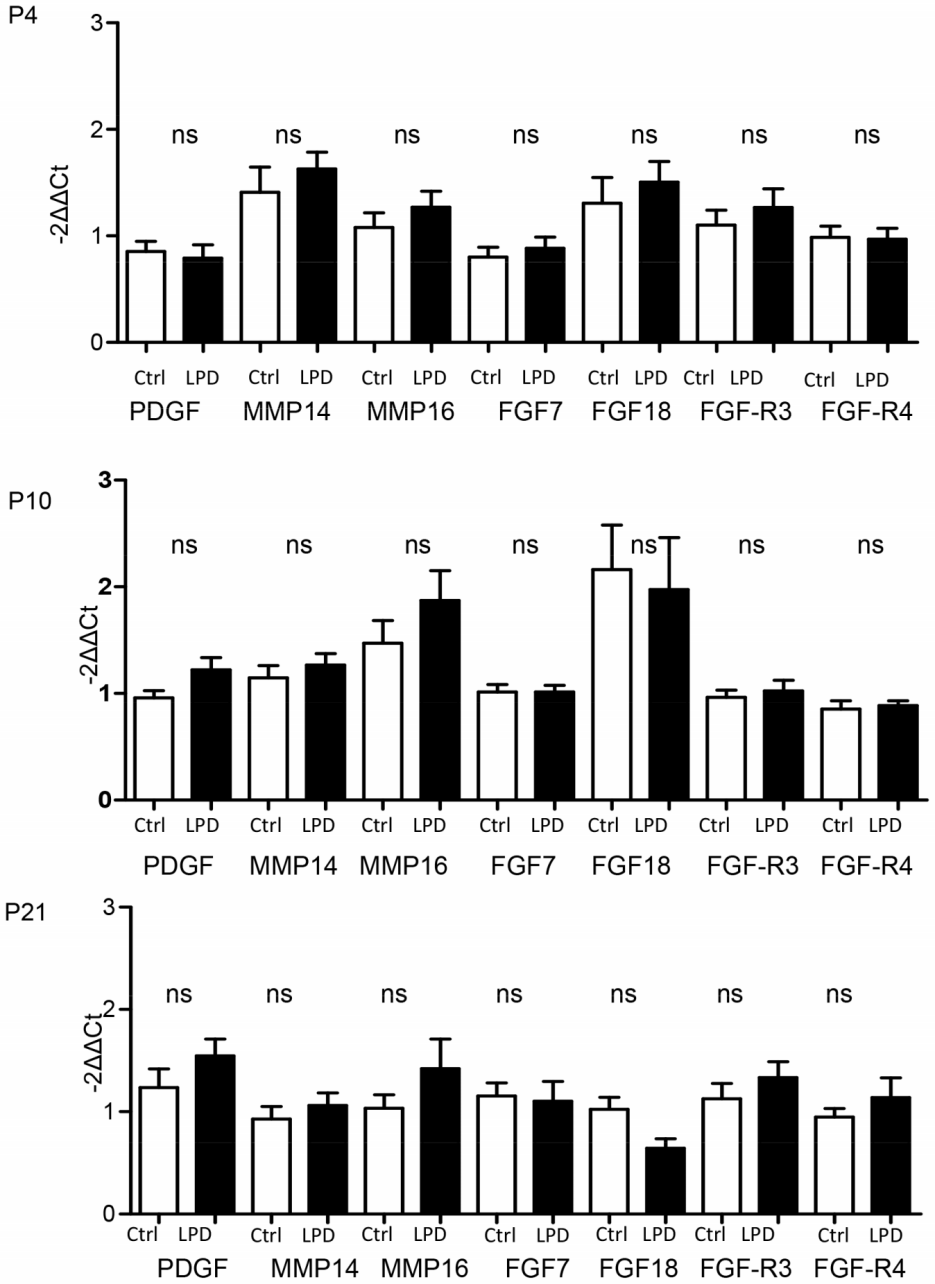
Effect of low protein diet-induced IUGR on mRNA expression of factors involved in alveolarization at day 4, 10 and 21. Expression of PDGF, MMP14 and 16, FGF7 and 18 and their receptors FGFR3 and 4 in neonatal rat lungs. n = 9–10 per group. mRNA expression was assessed by real-time PCR. The significance for each bar is indicated by *: p<0.05. Control vs. LPD; two-tailed Mann-Whitney test. Values are expressed as mean ± SEM.

**Figure 6:**
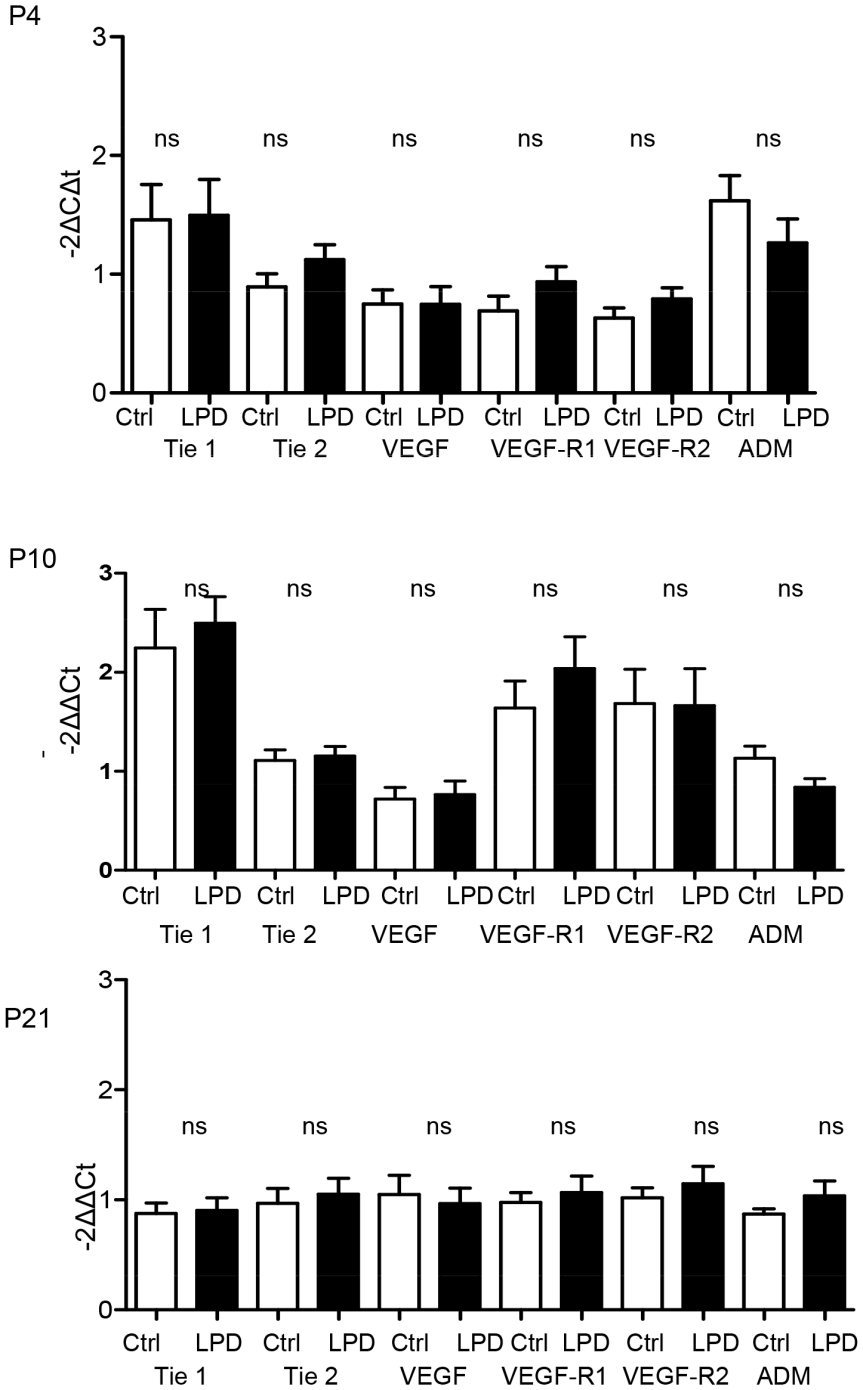
Effect of low protein diet-induced IUGR on mRNA expression of factors involved in angiogenesis at day 4, 10 and 21. Expression of Tie 1 and 2, VEGF and its two receptors (VEGF-R1 and 2) and ADM at P10 in neonatal rat lungs. n = 9–10 per group. mRNA expression was assessed by real-time PCR. The significance for each bar is indicated by *: p<0.05. Control vs. LPD; two-tailed Mann-Whitney test. Values are expressed as mean ± SEM.

mRNA levels of factors involved in alveologenesis were assessed on P4, P10 and P21 in rat pups. FGF7, FGF18, FGFR3 and -R4, MMP14 and 16 and PDGF-A expressions were not modified in the LPD group compared to the control group regardless of the time-points considered (Fig. 5). mRNA levels of genes involved in angiogenesis were assessed at the same time-points. In the LPD group, Tie 1 and 2, VEGF and its VEGFR1 and 2 receptors and adrenomedullin gene expressions were not modified regardless of the time-points considered (Fig. 6).

#### Gene expression in L-NAME-induced IUGR

**Figure 7:**
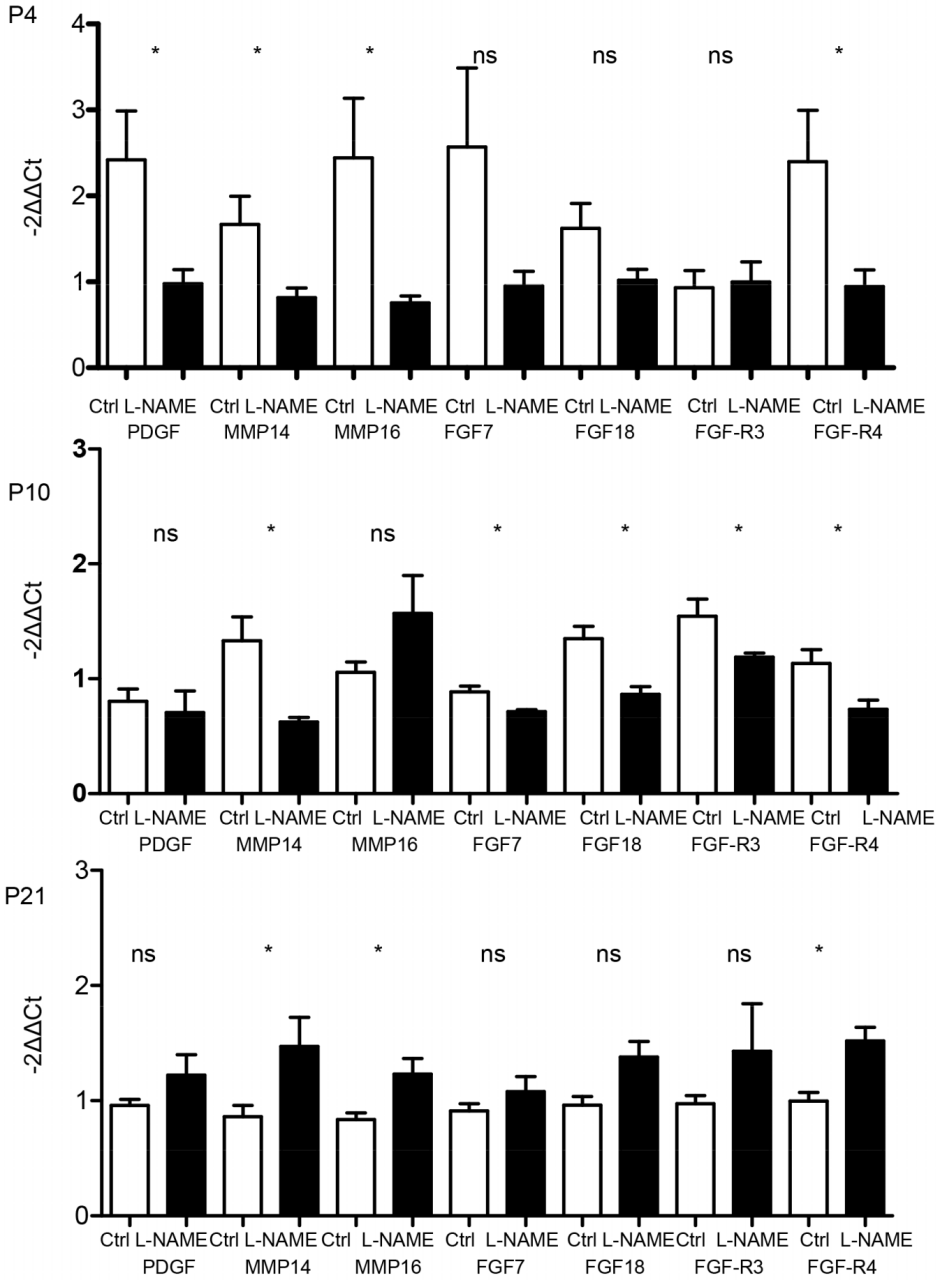
Effect of L-NAME-induced IUGR on mRNA expression of factors involved in alveolarization at day 4, 10 and 21. Expression of PDGF, MMP14 and 16, FGF7 and 18 and their receptors FGFR3 and 4 in neonatal rat lungs. n = 4–5 rat pups per group. mRNA expression was assessed by real-time PCR. The significance for each bar is indicated by *: p<0.05, control vs. L-NAME; two-tailed Mann-Whitney test. Values are expressed as mean ± SEM.

**Figure 8:**
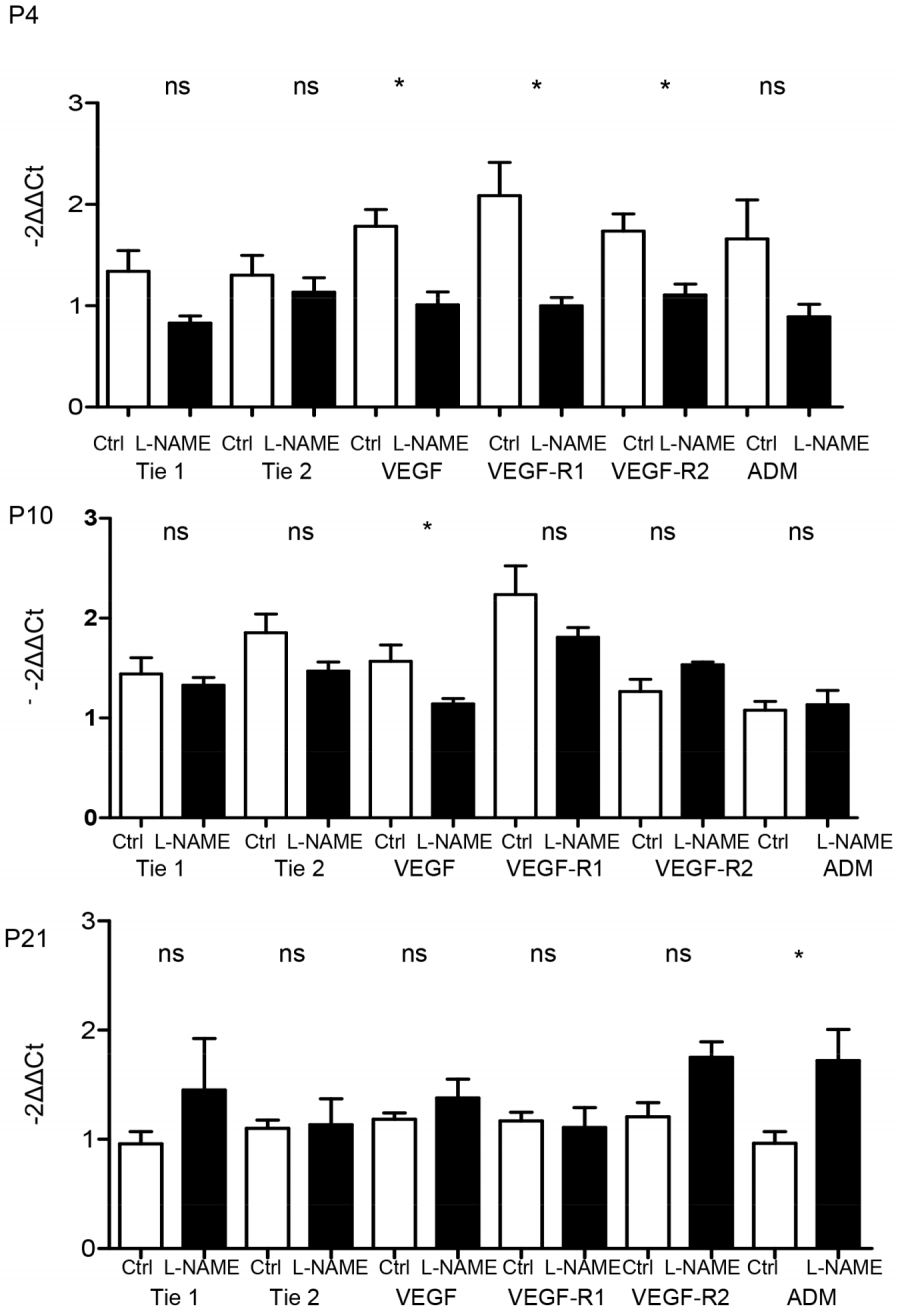
Effect of L-NAME-induced IUGR on mRNA expression of factors involved in angiogenesis at day 4, 10 and 21. Expression of Tie 1 and 2, VEGF and its two receptors (VEGFR1 and 2) and ADM in neonatal rat lungs. n = 4–5 rat pups per group. mRNA expression was assessed by real-time PCR. The significance for each bar is indicated by *: p<0.05, control vs. L-NAME; two-tailed Mann-Whitney test. Values are expressed as mean ± SEM.

mRNA levels of factors involved in alveologenesis and angiogenesis were assessed on P4, P10 and P21. On P4, MMP14, MMP16, FGFR4 and PDGF expressions were significantly decreased in the L-NAME group compared to the control group. Fibroblast growth factor 7, FGF18, FGF-R3 and -R4 and MMP14 expressions were significantly decreased in the L-NAME group compared to the control group on P10. Matrix metalloprotease 14, MMP16 and FGFR3 expressions were significantly increased on P21 in the L-NAME group compared to the control group (Fig. 7). Expression of VEGF, a gene involved in angiogenesis, was significantly decreased in the L-NAME group compared to the control group on P4 and P10 (Fig. 8). Moreover, on P4, the expression of VEGF receptors, VEGF-R1 and –R2 was significantly decreased in the L-NAME group. On P21, adrenomedullin expression was significantly increased in the L-NAME group compared to the control group.

## Discussion

This study, comprising complete structural analysis of lung tissue from rat pups at three key time-points of the alveolarization process, from the start of alveolarization on day 4, to the end of alveolarization on day 21 [16], [20], demonstrated that LPD-induced IUGR in rats induced arrested alveolarization similar to that observed in BPD. In this model, alveolarization was normal at the beginning of the alveolarization process, but became progressively impaired and markedly decreased at the end of the process. To our knowledge, this is the first study in rodents showing progressive impairment of alveolarization induced by conditions leading to IUGR. In contrast, the L-NAME model appears to be inappropriate to study long-term consequences of IUGR for lung development, as alveolarization was impaired at P4 but subsequently returned to normal by P21. This study also demonstrates major differences between the two IUGR models in terms of the expression of key genes known to be involved in alveolarization and lung angiogenesis.

In the L-NAME model, morphometric analysis revealed significant differences in alveolarization at the beginning of the alveolarization process, on P4, with correction on P21. These results are similar to previously published results in this model showing decreased alveolarization on P0 with a catch-up on P7 and P14 [11]. Although a different method was used to deliver L-NAME in our study [11], daily gavage versus subcutaneous pump, our study confirms that, in parallel to postnatal catch-up growth, lung development disorders present at birth in IUGR pups may be completely corrected in this model. The L-NAME model therefore appears to be inappropriate to evaluate the consequences of IUGR on alveolarization. A reversible effect of L-NAME model has been previously demonstrated: L-NAME infusion from embryonic day (E) 14 to E18 of gestation resulted in normal fetal and placental growth on E21. However, Neerhof showed that perfusion of L-NAME from day 14 to 18 of gestation only resulted in normal fetal and placental growth on P21, suggesting a reversible phenomenon [21]. Recent studies have also failed to demonstrate any long-term metabolic consequences in this model [15], questioning the value of this model to study the medium-term and long-term consequences of IUGR.

The experimental settings of the two models used in this study clearly varied in terms of the exposure window (last 4 days of gestation *vs.* throughout gestation) and the pathophysiological cause of low birth weight (Nitric Oxide synthase inhibition v*s.* malnutrition). L-NAME treatment administered at E17 to induce IUGR in rodents was first described 20 years ago [22]. Trials of earlier administration of L-NAME: from day 1 to day 18, resulted in a decreased number of implantation sites, decreased litter and placenta weights and an increased number of maternal deaths [23]. When administered at E12, L-NAME induced fetal resorptions and hind limb necrosis, which were not observed when it was administered after E15 [24]. Consequently, administration of L-NAME throughout gestation induces effects other than IUGR and, in order to induce IUGR, L-NAME treatment is therefore classically started in the last third of gestation. Although exposure time to each treatment could explain partly the differences observed between the 2 models, this study design nevertheless allows comparison of the pulmonary consequences of IUGR in pups of similar weights at P4.

Several reports suggest that LPD-induced IUGR is responsible for long-term metabolic consequences that are not reversible, even in adulthood: low muscle mass [25], adult-onset glucose intolerance [26], adult hypertension [27], and early aging [28]. In our study, in the LPD-induced IUGR model, all parameters quantifying alveolarization on P4 were equal to those observed in the control group, but indicated decreased alveolarization in the LPD group at P21: MLI, which is inversely correlated with alveolarization, was equal in the 2 groups at P4 and increased in the LPD group at P21; Sv(a,p) was identical at P4, but significantly decreased at P21 in the LPD group; RAC exhibited the same pattern and was also decreased at P21. Notably, LPD group pups regained normal weight at P21 and morphometric parameters can therefore be interpreted as reflecting a real reduction of alveolarization, independently of weight gain. In a previous study, we observed that morphometric parameters were closely correlated with body weight and differences between groups may be difficult to interpret when body weights are different [29]. These results suggest an arrest or at least a marked impairment of alveolar development. Consequently, this experimental model can be considered to very closely fit the characteristics of BPD, correlated with arrested alveolarization [1]. These results appear to be discordant with those previously published in a different rat strain with slightly different maternal diets, *eg* lower protein intake in LDP and in controls [30]. These authors did not observe any impaired alveolar development on P70, but based on a less complete morphometric analysis than that conducted in our study and, more surprisingly, increased alveolarization in the LPD group at P70.

Our study also investigated whether the gene expression of factors involved in alveolar and vascular development was affected by IUGR. Factors involved in alveolarization have been studied by three main approaches: 1) alveolarization study when these factors were not expressed in KO models [31] or by specific inhibition [32]; 2) expression of these factors during normal alveolarization [33]; 3) changes in the expression of these factors in models of postnatal insult leading to alveolarization disorders such as hyperoxia or mechanical ventilation [32]. In some cases, attempts to correct induced alveolarization disorders by administration of these factors were investigated, but with only partial success except for VEGF therapy [34]. Alveologenesis is a process coordinated by multiple interactions involving paracrine mechanisms between lung fibroblastic, epithelial, microvascular and extracellular matrix (ECM) components [16]. The factors studied here have been extensively described in this process. The role of antenatal events in the pathogenesis of alveolarization disorders has been well documented, especially in antenatal inflammation models [35]. However, alterations of factors involved in alveolarization have been less extensively studied in models and the results are fairly discordant. Intra-amniotic injection of group B streptococcus in macaques induced abnormal alveolar development with altered expressions of MMP2 and angiopoietin only [36]. Overall, the results are fairly discordant, as factors and their modifications vary from one model to another, highlighting the complexity of processes in which each factor is involved at a precise timing with precise interactions with other factors.

In the L-NAME model, factors previously shown to be involved in alveolarization, MMP14, FGFR3 and 4, FGF18 and 7, were significantly decreased on P4 and/or P10, but this decrease was not associated with impaired alveolarization at P10. Interestingly, L-NAME, given from E10 to E20 in pregnant Sprague-Dawley rats via an osmotic pump, decreased MMP14 expression in the uterine vessel wall on E20 [37]. Whether this effect on MMP14 is also observed in the fetus and during the first days of life has yet to be studied. Surprisingly, MMP14 expression was significantly increased in the L-NAME group on P21, at a time when the alveolarization process is theoretically completed and the decreased alveolarization observed at P4 has been corrected much earlier as observed in our study at P10 and in other studies at P7 [11]. Similar surprising results were observed for VEGF expression. VEGF increased during alveolarization, and decreased when alveolarization was impaired. VEGF inhibition decreased alveolarization and VEGF administration restored alveolarization, even after the end of the alveolarization process [34]. In our study, antenatal L-NAME treatment induced a sustained decrease of VEGF and its receptor expressions (P4 and/or P10) during the alveolarization process and in the period during which alveolarization can be assumed to have been restored in this model. Finally, none of the factors usually described as essential for alveolarization were modified in the LPD model. These results raise the following question: when the alveolarization process is impaired antenatally, does it respond to regulation factors other than those involved in the normal process and in postnatal models of impaired alveolarization in rat pups?

It has now been clearly established that the developing fetus perceives the environment during specific windows of sensitivity and optimizes future metabolic responses by reprogramming its genome. This reprogramming promotes early survival and reproductive success, but potentially causes a predisposition to disease in later life [10], that only develops when an additional stress occurs subsequently (preterm birth, obesity or high-fat diet). The hypothesis of the fetal or “early” origin of adult disease was originally proposed by Barker, who stated that environmental factors, particularly nutrition, act in early life to program risks for metabolic syndrome, hypertension, insulin resistance and obesity. Lung consequences of IUGR have now also been clearly demonstrated with an increased incidence of BPD [3] and long-term impairment of lung function [8]. Our results suggest that a compromised intrauterine environment could disrupt normal fetal lung development by pathways other than those described in classical models of altered alveolarization. When a modification of programming occurs, involvement of growth factors is inconstant as demonstrated for other organs in IUGR models. Microarray analysis in LPD-induced IUGR in rat showed that only 5 of the 54 crucial factors for kidney development were significantly modified [14]. Proteomic studies in IUGR piglets have shown that, in addition to a significant reduction in small intestine, liver and skeletal muscle masses, IUGR was also associated with an alteration of proteins known to regulate cellular signalling and intermediary metabolism, resulting in increased proteolysis and reduced polypeptide synthesis [38]. However, the consequences on lung development specifically related to intrauterine growth restriction need to be more thoroughly investigated.

In conclusion, our results demonstrate that two models of IUGR, L-NAME and LPD, have very different impacts on lung development and gene expression patterns of key factors involved in lung development. Only the LPD model closely fits with IUGR-induced alveolarization disorder. The absence of involvement of key factors of alveolarization despite abnormal lung development raises the issue of the role of other regulators. Morbidity induced by IUGR should be analyzed in terms of its pathophysiological cause rather than low birth weight *per se* and LPD-induced IUGR may therefore constitute a useful tool for an experimental approach to IUGR-induced BPD.

## Supporting Information

Table S1 **PCR primers for quantitative real-time PCR.**
(DOC)

Table S2 **Morphometric analysis of the lung of control and low protein diet-induced intrauterine growth restriction groups.** Significance for each time-point is indicated by symbols; two-tailed Mann-Whitney test (p<0.05). Values are expressed as mean ± SEM. n = 5 animals per group.
(DOC)

Table S3 **Morphometric analysis of the lung of control and L-NAME- induced intrauterine growth restriction groups.** Significance for each time-point is indicated by symbols; two-tailed Mann-Whitney test (p<0.05). Values are expressed as mean ± SEM. n = 5 animals per group.
(DOC)
