# Supplementary material for: Effect of Two Models of Intrauterine Growth Restriction on Alveolarization in Rat Lungs: Morphometric and Gene Expression Analysis
Source: PLoS One. 2013 Nov 21;8(11):e78326. doi: 10.1371/journal.pone.0078326 (PMC3836790; doi:10.1371/journal.pone.0078326)
Supplement: Table S1 — PCR primers for quantitative real-time PCR. (DOC) [file pone.0078326.s001.doc]

***Table S*** 1: PCR primers for quantitative real-time PCR

| Name | NCBI reference sequence | Forward | Reverse |
| --- | --- | --- | --- |
| FGF7 | NM_022182 | CTGTGGCAGTTGGAATTGTG | TCATTGCATTCTTTCTTTGCAT |
| FGF18 | NM_019199 | CCTGCACTTGCCTGTGTTTA | CCGAGTCTGGTTCTCCACAT |
| FGFR3 | NM_053429 | AACAGATGCTCCGTCCTCAG | AGCGGAAGCGTACAGTGTTT |
| FGFR4 | NM_001109904 | GGATGACTCCTTACCCTCCA | GGTGTGTCCAGTAAGGTGCTT |
| MMP14 | NM_031056 | TACCTACCTCCAGGGGACCT | GCCTCATAGCCTTCATCGTG |
| MMP16 | NM_080776 | GAGCTGGGACATGCTCTAGG | GAGGGATCTTGTCAGGTGGA |
| Tie 1 | NM_053545 | AAGGTCACACACACGGTGAA | TGGTGGCTGTACATTTTGGA |
| Tie 2 | NM_001105737 | AGAAGTGCATCTGCCTCACG | TTCACATCTCCGGACAATCA |
| PDGF-A | NM_012801 | ATGCCTTGGAGACAAACCTG | GGGAATGGCTTCCTCAATACT |
| ADM | NM_012715 | GGGCTCGTTGATGAGAAGAC | GGGCTGTGCTCTGAGTGCT |
| VEGF-A | NM_031836 | CGTCTACCAGCGCAGCTATTG | CACACAGGACGGCTTGAAGAT |
| VEGF-R2 | NM_013062 | TTGGCAAATACAACCCTTCAGAT | CACTCAGTCACCAACACCCTTTC |
| VEGF-R1 | NM_005111 | AGCGCATGATGGTCATAGAA | TTGGCACATCTGTGACGTAAA |
