# Supplementary material for: Effect of Two Models of Intrauterine Growth Restriction on Alveolarization in Rat Lungs: Morphometric and Gene Expression Analysis
Source: PLoS One. 2013 Nov 21;8(11):e78326. doi: 10.1371/journal.pone.0078326 (PMC3836790; doi:10.1371/journal.pone.0078326)
Supplement: Table S2 — Morphometric analysis of the lung of control and low protein diet-induced intrauterine growth restriction groups. Significance for each time-point is indicated by symbols; two-tailed Mann-Whitney test (p<0.05). Values are expressed as mean ± SEM. n = 5 animals per group. (DOC) [file pone.0078326.s002.doc]

Table S 2: Morphometric analysis of the lung of control and low protein diet-induced intrauterine growth restriction groups.

| Day | P4 (n=5) | | P10 (n=5) | | | | P21 (n=5) | | |
| --- | --- | --- | --- | --- | --- | --- | --- | --- | --- |
| Group | Control | LPD | Control | | LPD | | Control | | LPD |
| Alveolar surface area | | | | | | | | | |
| Sv(a,p) (cm²/cm^3^) | 96.27±12.28 | 88.29±8.00 | 110.01±10.07 | 88.17±10.82 § | | 112.79±9.28 | | 86.67±9.4 ¥ | |
| Sa (cm²) | 56.04±10.27 | 34.162±4.94 * | 121.24±26.15 | 64.46±14.8 § | | 197.38±33.77 | | 141.75±23.68 ¥ | |
| Alveolar parenchyma | | | | | | | | | |
| Vvp (%) | 0.91±0.04 | 0.91 ±0.02 | 0.93±0.02 | 0.91 ±0.002 | | 0.95±0.012 | | 0.94±0.017 | |
| Mean Liner Intercept | | | | | | | | | |
| MLI (cm) | 0.04±0.006 | 0.05±0.006 | 0.039±0.004 | 0.05±0.007 § | | 0.037±0.003 | | 0.05±0.005 ¥ | |
| Radial alveolar count | | | | | | | | | |
| n= | 4.66±0.92 | 3.63±0.96 | 5.5±1.38 | 4.4±2.08 § | | 8.78±1.7 | | 4.66±0.94 ¥ | |

* Significantly different at P4 between low protein diet (LPD) and control groups (p≤0.05)

§ Significantly different at P10 between LPD and control groups (p≤0.05)

¥ Significantly different at P21 between LPD and control groups (p≤0.05)

Data are expressed as mean ± SEM. Statistical analysis was performed with Mann-Whitney test

Abbreviations: n=number; Sv(a,p), alveolar surface density; Vvp, volumetric density of lung alveolar parenchyma; MLI, mean linear intercept; Sa, absolute surface area of airspaces.
