# Supplementary material for: Effect of Two Models of Intrauterine Growth Restriction on Alveolarization in Rat Lungs: Morphometric and Gene Expression Analysis
Source: PLoS One. 2013 Nov 21;8(11):e78326. doi: 10.1371/journal.pone.0078326 (PMC3836790; doi:10.1371/journal.pone.0078326)
Supplement: Table S3 — Morphometric analysis of the lung of control and L-NAME- induced intrauterine growth restriction groups. Significance for each time-point is indicated by symbols; two-tailed Mann-Whitney test (p<0.05). Values are expressed as mean ± SEM. n = 5 animals per group. (DOC) [file pone.0078326.s003.doc]

Table S 3: Morphometric analysis of the lung of control and L-NAME- induced intrauterine growth restriction groups.

| Day | P4 (n=5) | | | P10 (n=5) | | | P21 (n=5) | |
| --- | --- | --- | --- | --- | --- | --- | --- | --- |
| Group | Control | L-NAME | | Control | | L-NAME | Control | L-NAME |
| Alveolar surface area | | | | | | | | |
| Sv(a,p) (cm²/cm^3^) | 80.6±15.3 | 58.62±5.31 * | 85.58±8.78 | | 78.35±21.24 | | 96.97±7.05 | 102.08±13.75 |
| Sa (cm²) | 52.35±9.53 | 28.43±5.44* | 109.16±5.57 | | 67.06±24.52 § | | 163.18±41.58 | 216.8±16.89 |
| Alveolar parenchyma | | | | | | | | |
| Vvp (%) | 0.92±0.017 | 0.9 ±0.002 | 0.92±0.021 | | 0.85 ±0.114 | | 0.92±0.03 | 0.95 ±0.018 |
| Mean Liner Intercept | | | | | | | | |
| MLI (cm) | 0.06±0.009 | 0.08±0.007 * | | 0.05±0.005 | | 0.06±0.01 | 0.04±0.003 | 0.04±0.005 |
| Radial alveolar count | | | | | | | | |
| n= | 4.4±1.02 | 2.7±0.58 * | 7.01±1.44 | | 4.57±1.38 § | | 9.08±1.5 | 8.38±1.84 |

* Significantly different at P4 between Control and L-NAME group (p≤0.05)

§ Significantly different at P10 between Control and L-NAME group (p≤0.05)

Data are expressed as mean ± SEM. Statistical analysis was performed with Mann-Whitney test

Abbreviations: n=number; Sv(a,p) alveolar surface density; Vvp, volumetric density of lung alveolar parenchyma; MLI, mean linear intercept; Sa, absolute surface area of airspaces
